# Using Culture and Whole Genome Sequencing to Assess Sterilization to Reduce Bacterial Contamination of Ventilator Heater Wires

**DOI:** 10.1017/ash.2025.237

**Published:** 2025-09-24

**Authors:** Rachel Sidebottom, William Johnson, April Vigil, Daryl Domman, Meghan Brett

**Affiliations:** 1 University of New Mexico School of Medicine; 2 UNM Hospital; 3 University of New Mexico

## Abstract

Ventilator-associated events (VAEs), including ventilator-associated pneumonia (VAP), are important among hospitalized patients due to their high morbidity, mortality, and associated costs. These infections are frequently caused by multidrug-resistant ESKAPE pathogens (Enterococcus, Staphylococcus, Klebsiella, Acinetobacter, Pseudomonas, and Enterobacter), which are known for their antibiotic resistance.

Heater wires used in mechanical ventilators regulate air humidity and temperature which prevents complications when the upper airway is bypassed. However, because these are in direct contact with the air supplied to patients, they can become sources of infection and reservoirs for antimicrobial-resistant organisms.

At a hospital in New Mexico, we transitioned from using low-level disinfectant wipes to sterile processing for heater wires. The dry climate in New Mexico accelerates the evaporation of disinfectants, reducing their effectiveness by shortening their contact time. Additionally, achieving full surface coverage with disinfectant wipes is difficult, compromising sterilization effectiveness.

To address these challenges, we implemented a protocol to send heater wire probes to sterile processing for sterilization. We evaluated the impact of this change by comparing the prescence of bacteria on the probes before and after sterilization. Swabs from heater wire prongs were cultured and sequenced using Oxford Nanopore Technology. Metagenomic sequencing and analysis was also performed.

Before the new protocol, we swabbed 19 clean probes and 11 used probes. Bacterial DNA was detected on all clean probes and bacterial growth found on 42% of clean probe cultures. Of these, 63% were positive for ESKAPE pathogens, with five out of eight probes showing all ESKAPE species, and three probes lacking only Enterobacter. Additionally, all of the clean probe cultures were positive for Stenotrophomonas, another well known multi-drug resistant pathogen. After the autoclaving protocol was implemented, no bacterial growth was observed cultures (72 hours) of freshly sterilized probes.

In conclusion, sterilization significantly improved the cleanliness of heater wires over use of disinfectant wipes. This improved sterilization protocol is expected to reduce the risk of infection transmission and the incidence of VAEs, thereby improving patient safety and outcomes.

